# The discriminative ability of FRAX, the WHO algorithm, to identify women with prevalent asymptomatic vertebral fractures: a cross-sectional study

**DOI:** 10.1186/1471-2474-15-365

**Published:** 2014-11-04

**Authors:** Abdellah El Maghraoui, Siham Sadni, Nabil Jbili, Asmaa Rezqi, Aziza Mounach, Imad Ghozlani

**Affiliations:** Rheumatology department, Military Hospital Mohammed V, PO Box: 1018, Rabat, Morocco

**Keywords:** FRAX, Bone density, Female, Vertebral fractures, VFA, DXA, Bone, Osteoporosis, Postmenopausal, Menopause, Risk factors, Sensitivity and specificity

## Abstract

**Background:**

A Moroccan model for the FRAX tool to determine the absolute risk of osteoporotic fracture at 10 years has been established recently. The study aimed to assess the discriminative capacity of FRAX in identifying women with prevalent asymptomatic vertebral fractures (VFs).

**Methods:**

We enrolled in this cross-sectional study 908 post-menopausal women with a mean age of 60.9 years ±7.7 (50 to 91) with no prior known diagnosis of osteoporosis. Subjects were recruited from asymptomatic women selected from the general population. Lateral VFA images and scans of the lumbar spine and proximal femur were obtained using a GE Healthcare Lunar Prodigy densitometer. VFs were defined using a combination of Genantsemiquantitative (SQ) approach and morphometry. We calculated the absolute risk of major fracture and hip fracture with and without bone mineral density (BMD)using the FRAX website.The overall discriminative value of the different risk scores was assessed by calculating the areas under the ROC curve (AUC).

**Results:**

VFA images showed that 179 of the participants (19.7%) had at least one grade 2/3 VF. The group of women with VFs had a statistically significant higher FRAX scores for major and hip fractures with and without BMD, and lower weight, height, and lumbar spine and hip BMD and T-scores than those without a VFA-identified VF. The AUC ROC of FRAX for major fracture without BMD was 0.757 (CI 95%; 0.718-0.797) and 0.736 (CI 95%; 0.695-0.777) with BMD, being 0.756 (CI 95%; 0.716-0.796) and 0.747 (CI 95%; 0.709-0.785), respectively for FRAX hip fracture without and with BMD. The AUC ROC of lumbar spine T-score and femoral neck T-score were 0.660 (CI 95%; 0.611-0.708) and 0.707 (CI 95%; 0.664-0.751) respectively.

**Conclusion:**

In asymptomatic post-menopausal women, the FRAX risk for major fracture without BMD had a better discriminative capacity in identifying the women with prevalent VFs than lumbar spine and femoral neck T-scores suggesting its usefulness in identifying women in whom VFA could be indicated.

**Electronic supplementary material:**

The online version of this article (doi:10.1186/1471-2474-15-365) contains supplementary material, which is available to authorized users.

## Background

Although assessing bone mass with dual-energy X-ray absorptiometry (DXA) is the gold standard for osteoporosis diagnosis, studies have shown that most fractures occur in individuals with a BMD T-score above the WHO operational threshold for osteoporosis[1]. Recently, the use of clinical risk factors (CRFs) has been shown to enhance the performance of BMD in the prediction of hip and major osteoporotic fractures. In addition to a prior fragility fracture, CRFs include age, sex, body mass index (BMI), use of glucocorticoids, secondary osteoporosis, rheumatoid arthritis, parental history of hip fracture, current smoking, and alcohol intake of three or more units/day. The WHO fracture risk assessment tool (FRAX) allows for estimation of individual 10-year major osteoporotic and hip fracture probabilities[2].

Vertebral fractures (VFs) are the most common type of osteoporotic fractures in older adults. It has been shown that VFs are usually asymptomatic (only one fourth to one third of these fractures come to medical attention)[3] and that women with a VF are four to five times more likely to suffer another VF and are also at increased risk for hip fracture and other nonspine fractures compared with women without a VF;[4, 5] thus their detection remains an important challenge for clinicians. Moreover, radiographically detected VFs are associated with reduced quality of life, increased morbidity and mortality[6]. Consequently, the identification of asymptomatic VFs is of primordial importance especially in patients without densitometric osteoporosis, a common situation where all experts agree to recommend treatment[7, 8]. Recently, Vertebral fracture assessment (VFA), which is a method for imaging the thoraco-lumbar spine using bone densitometers[9] has been showed to have good accuracy and reliability. It can easily be performed at the time of bone mineral density (BMD) measurement, allowing integration of BMD and VF information in the clinical care of patients evaluated for osteoporosis[1]. Advantages of VFA compared with spine radiographs include greater patient convenience (VFA can be done in association with BMD testing by DXA), smaller dose of ionizing radiation, and lower cost. Previous studies report that, using VFA, around 90–95% of vertebra are interpretable[10–12]. The majority of uninterpretable vertebra occur above T7,[13, 14] where the prevalence of fracture is low, preserving the negative predictive value of VFA[15].

The performance characteristics of the FRAX tool have been validated in many independent cohorts[16].However, most if not all of these cohorts concerned elderly women, usually over the age of 65 and have mainly focused on hip fractures[17, 18]. There is some uncertainty as to whether this screening tool would have the same performances in younger postmenopausal women and in identifying asymptomatic VFs. Recently, using VFA, we found that 19.7% of a cohort of asymptomatic women show evidence of moderate/severe VFs[19]. As a FRAX model was developed recently for Morocco based on a large epidemiological study of hip fractures,[20] we aimed in the present study to evaluate the performance of FRAX scores in comparison with BMD measurement in identifying women with prevalent asymptomatic VFs.

## Methods

### Subjects

This was a cross-sectional study conducted from june 2010 to march 2012 with menopausal women 50 years old and over, living in the region of Rabat, recruited from the general population through advertisements and “word of mouth” in local hospitals. Both the rationale and the study design have been described in details elsewhere[19]. Briefly, nine hundred and eight consecutive women who had no previous diagnosis of osteoporosis were entered into the study. General exclusion criteria were non-Caucasian origin and diseases, drugs, and other major determinants known to affect bone metabolism. Thus, we excluded subjects with gastrectomy, intestinal resection, recent hyperthyroidism or hyperparathyroidism, recent severe immobilization, treatment with corticosteroids (more than 3 months), breast cancer or aromatase inhibitors. Our institutional review board approved this study (Comité d’éthique et de recherche de l’hôpital Militaire Mohammed V). The procedures of the study were in accordance with the Declaration of Helsinki, and formal local ethics committee approval was obtained for the study (Comité d’éthique de la Faculté de Médecine et de Pharmacie de Rabat). All the participants gave an informed and written consent. Each subject completed a standardized questionnaire designed to document putative risk factors of osteoporosis. History of fractures and lifestyle habits (alcohol consumption, gymnastics or jogging/walking, smoking) were also recorded. Menstrual and reproductive history were assessed: all patients were menopausal since at least one year. Height and weight were measured in our centre before DXA measurement, in light indoor clothes without shoes. Body mass index (BMI) was calculated by dividing weight in kilograms by height in meters squared.

### BMD measurement

BMD was determined by a Lunar Prodigy Vision DXA system (GE Healthcare, Madison, WI). The DXA scans were obtained by standard procedures supplied by the manufacturer for scanning and analysis. All BMD measurements were carried out by 2 experienced technicians. Daily quality control was carried out by measurement of a Lunar phantom. At the time of the study, phantom measurements showed stable results. The phantom precision expressed as the coefficient of variation percentage was 0.08. Moreover, reproducibility has been assessed in clinical practice and showed a smallest detectable difference of 0.04 g/cm^2^ (spine) and 0.02 (hips)[21, 22]. Patient BMD was measured at the lumbar spine (anteroposterior projection at L1-L4) and at the femurs (i.e., femoral neck, trochanter, and total hip). The World Health Organization (WHO) classification system was applied, defining osteoporosis as T-score ≤ -2.5 and osteopenia as -2.5 < T-score < -1. Study participants were categorized by the lowest T-score of the L1–4 lumbar spine, femur neck, or total femur.

### Vertebral fracture assessment

All patients had a VFA imaging at the same time of their BMD evaluation and all VFA scans were studied in a separate occasion to assess the presence of VFs by the same reader (IG) who was blinded to patient clinical data. VFs was classified using a combination of Genant[23] semiquantitative (SQ) approach and morphometry in the following manner: each VFA image was inspected visually by one clinician (IG), who is an experienced reader of VFA, to decide whether it contained a fracture in any of the visualized vertebrae and assigned a grade based on Genant SQ scale, where grade 1 (mild) fracture is a reduction in vertebral height of 20-25%, grade 2 (moderate) a reduction of 26-40%, and grade 3 (severe) a reduction of over 40%. In case of doubt regarding fracture grade, the vertebrae in question was measured using built-in morphometry. Automatic vertebral recognition by the software was used. Positioning of the six morphometry points was modified only when the software failed to correctly recognize vertebral heights.

### Statistical analysis

Results are presented as means (SD) and categorical variables are expressed as frequencies. To compare patients with and without VFs, chi-square test and analysis of variance ANOVA were used firstly. To assess and compare the discriminatory value for fracture of FRAX and BMD in identifying women with VFs, we used the Moroccan model for the FRAX algorithm available at http://www.shef.ac.uk/FRAX to calculate individual 10-year probability of hip and major osteoporotic fractures based on each woman’s clinical characteristics with or without hip BMD measurement value. We assessed the overall discriminative value of the different risk scores by calculating the areas under the ROC curve (AUC). Higher AUC values represent better prediction with the models. We also assessed the sensitivity (proportion of women with VFs who had been classified as high risk) and specificity (proportion for women without VFs who had not been classified as high risk) of each risk score for various definitions of the high-risk group based on different cut-offs. We also calculated the corresponding positive and negative predictive values and the positive likelihood ratios. The level for significance was taken as p ≤0.05. Excel 2007 and SPSS 20.0 were used for statistical analysis and STATA 12.0 was used for ROC curves comparison (Stata Core, College Station, Texas). The manuscript has adhered to the STROBE guidelines for observational studies (Additional file1).

### Ethics committee

The local ethics committee of our hospital approved this study.

## Results

### Patient demographics

In this series of 908 women, the mean ± SD (range) age, weight and BMI were 60.9 ± 7.7 (50 to 91) years, 73.2 ± 13.2 (35 to 150) kgs and 29.8 ± 5.3 (14.5 to 50.8) kg/m^2^, respectively (Table 1). According to the WHO classification, 283 had osteoporosis (31.2%) and 402 had osteopenia (44.3%). Only 4 women (0.4%) were current smokers. One hundred and eighteen (12.9%) women reported a history of traumatic peripheral fracture before the age of 50. None of women reported a low impact fracture after 50.

**Table 1:** **Comparison between patients with and without vertebral fractures**

	Group 0: Women without vertebral fractures N = 526	Group 1: Women with grade 1 vertebral fractures N = 203	Group 2: Women with grade 2 or 3 vertebral fractures N = 179	P-value 0 vs 2	P-value 0 vs 1	P-value 1 vs 2
Age (yrs): mean (SD)	59.1 (7.1)	60.7 (7.2)	66.5 (7.7)	<0.0001	0.037	<0.0001
Weight (Kgs): mean (SD)	74.4 (13.0)	74.5 (14.3)	68.5 (11.4)	<0.0001	NS	<0.0001
Height (m): mean (SD)	1.57 (0.06)	1.55 (0.06)	1.55 (0.06)	<0.0001	<0.0001	NS
Body Mass Index (Kg/m^2^): mean (SD)	30.0 (4.0)	30.7 (4.1)	28.4 (3.4)	<0.001	NS	<0.0001
Number of parity: mean (SD)	4.8 (2.3)	5.1 (2.5)	5.8 (3.1)	<0.0001	NS	NS
Years since menopause: mean (SD)	11.0 (8.2)	12.4 (8.5)	17.5 (9.4)	<0.0001	NS	<0.0001
Lumbar spine BMD (g/cm^2^): mean (SD)	0.998 (0.15)	0.945 (0.16)	0.873 (0.14)	<0.0001	<0.0001	<0.0001
Lumbar spine T-score: mean (SD)	-1.3 (1.2)	-1.7 (1.3)	-2.4 (1.2)	<0.0001	0.025	<0.0001
Femoral neck BMD (g/cm^2^): mean (SD)	0.925 (0.14)	0.878 (0.13)	0.830 (0.12)	<0.0001	0.002	0.049
Femoral neck T-score: mean (SD)	-0.7 (1.0)	-1.2 (1.1)	-1.6 (1.0)	<0.0001	0.003	0.003
Total hip BMD (g/cm^2^): mean (SD)	0.930 (0.14)	0.866 (0.13)	0.815 (0.12)	<0.0001	0.003	0.002
Total hip T-score: mean (SD)	-0.7 (1.0)	-1.2 (1.1)	-1.6 (1.0)	<0.0001	0.003	0.002
T ≤ -2.5 at anysite: n(%)	109 (20.7)	77 (37.9)	98 (54.7)	<0.0001	<0.0001	<0.0001
FRAX major fracture without BMD	2.1 (1.2)	2.2 (1.2)	3.5 (1.7)	<0.0001	NS	<0.0001
FRAX hip fracture without BMD	0.4 (0.5)	0.4 (0.4)	1.0 (0.8)	<0.0001	NS	<0.0001
FRAX major fracture with BMD	2.2 (3.1)	2.5 (1.5)	3.4 (1.7)	<0.0001	NS	0.002
FRAX hip fracture with BMD	0.4 (1.9)	0.5 (0.7)	0.9 (1.0)	<0.001	NS	0.024

Data as mean (SD) or number (percent). We performed pairwise comparisons among the 3 groups using the Bonferoni test after an analysis of variance ANOVA.

### Vertebral visualization and fracture identification on VFA

In these 908 women, 88.7% of vertebrae from T4–L4 and 99% from T8–L4 were adequately visualized on VFA. The percentage of vertebrae not visualized at T4, T5, and T6 levels was 74.1%, 49.2, and 16.1% respectively. VFs were identified in 382 (42.1%): 203 (22.4%) had grade 1 and 179 (19.7%) had grade 2 or 3. The group of women with moderate/severe VFs had a statistically significant higher age, parities, FRAX risk for major fractures and hip fractures with and without BMD and lower weight, height, and lumbar spine and total hip BMD and T-scores than those without a VFA-identified vertebral fracture (Table 1).

**Figure 1:**
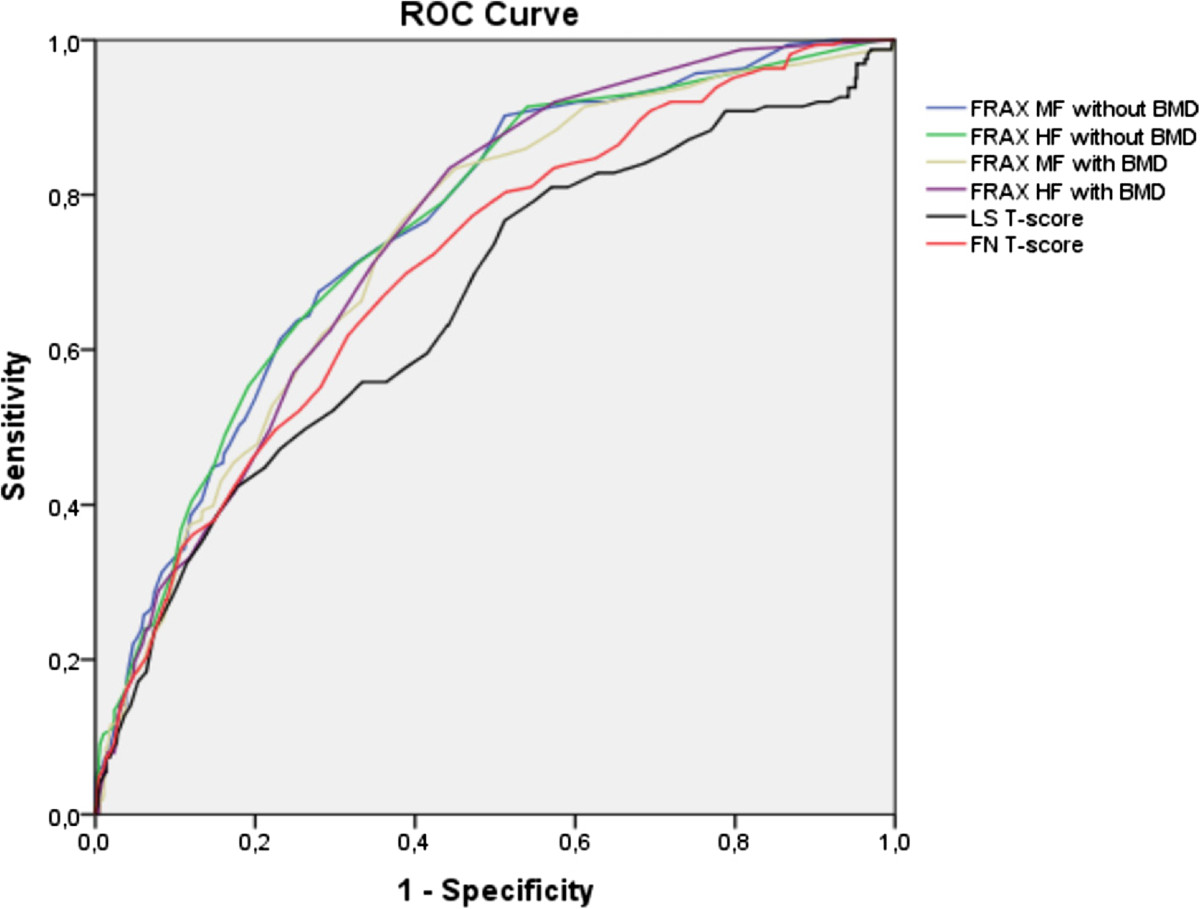
**Receiver Operating Characteristic (ROC) curves for prediction of vertebral fractures using FRAX tools and T-scores.** *MF: major fracture; HF: hip fracture, LS: lumbar spine; FN: femoral neck.

The AUCs are reported in Table 2 and depicted in Figure 1. A model with no utility in predicting fracture would have an AUC of 0.50 (i.e., no better than flipping a coin or chance alone); AUC was greater than 0.50 for all models. The highest AUC was observed for FRAX risk without BMD which was significantly higher than the AUC for lumbar spine (p = 0.0005) and femoral neck (p = 0.048) T-scores. Analysis did not show significant improvement when parity was added to FRAX risk with or without BMD. None of our subjects had a FRAX risk with or without BMD ≥20%.

**Table 2:** **Area Under Curve (AUC) of Receiver Operating Characteristics (ROC) for prediction of vertebral fractures using FRAX tools and T-scores**

	VFs grade 2/3		VFs grade 1	
	AUC	p	AUC	p
FRAX major fracture without BMD	0.757 (0.718 – 0.797)	<0.0001	0.637 (0.591 – 0.683)	<0.0001
FRAX hip fracture without BMD	0.756 (0.716 – 0.796)	<0.0001	0.631 (0.585 – 0.677)	<0.0001
FRAX major fracture with BMD	0.736 (0.695 – 0.777)	<0.0001	0.662 (0.618 – 0.707)	<0.0001
FRAX hip fracture with BMD	0.748 (0.710 – 0.786)	<0.0001	0.662 (0.618 – 0.706)	<0.0001
Lumbar spine T-score	0.660 (0.611 – 0.708)*	<0.0001	0.610 (0.569 – 0.650)	<0.0001
Femoral neck T-score	0.707 (0.664 – 0.751)*	<0.0001	0.658 (0.621 – 0.695)	<0.0001
FRAX major fracture without BMD with parity	0.697 (0.643 – 0.751)*	<0.0001	0.632 (0.585 – 0.678)	<0.0001

*Indicates p < 0.05 in comparison with the AUC of ROC of FRAX major fracture without BMD.

We then compared the sensitivity for fracture of FRAX risk (major fractures without BMD) and lumbar spine and femoral neck T-scores for various definitions of the high risk group based on percentile of their distribution in the study population (Table 3). If the cut-off for major fractures without BMDFRAX risk is set, for instance, at 3, the sensitivity is approximately equal to 57%, specificity to 78%, the positive predictive value to 40% and the positive likelihood ratio to 2.68.

**Table 3:** **Sensitivity, specificity, positive and negative predictive values and corresponding cut-offs of lumbar spine and femoral neck T-scores and FRAX major fracture model without BMD for the prediction of grade 2/3 vertebral fractures**

FRAX major fractures without BMD						Lumbar spine T-score						Femoral neck T-score					
Cut-off	Se	Sp	PPV	NPV	PLR	Cut-off	Se	Sp	PPV	NPV	PLR	Cut-off	Se	Sp	PPV	NPV	PLR
≥1%	99.4	13.4	21.8	98.9	1,15	≤-1	82.8	35.2	23.7	89.4	1,28	≤-1	81.0	45.5	26.5	90.8	1,49
≥2%	84.7	51.6	29.8	93.3	1,75	≤-2	57.7	61.3	26.6	85.6	1,49	≤-2	46.0	80.3	36.2	86.0	2,34
≥3%	57.1	78.7	39.4	88.3	2,68	≤-2.5	47.2	76.9	33.2	85.7	2,04	≤-2.5	27.6	91.2	43.3	83.8	3,14
≥4%	34.4	88.8	42.7	84.8	3,07	≤-3	32.5	88.5	40.8	84.4	2,83	≤-3	13.5	96.9	51.2	82.2	4,35

Se: sensitivity, Sp: specificity, PPV: positive predictive value, NPV: negative predictive value, PLR: positive likelihood ratio.

## Discussion

This study shows that FRAX risk with and without BMD can predict prevalent asymptomatic osteoporotic fractures in low risk postmenopausal women recruited from general population. In this population, ROC c-statistical analysis showed that the performance of FRAX risk without BMD was better than that of lumbar spine or femoral neck T-scores. Analysis of the BMD with the DXA technique for the axial skeleton has traditionally been considered as the best predictive test known to determine fragility VFs. Moreover, the strategy of intervention for VFs prevention in medical practice has been based on this test until the appearance of the importance of other risk factors for fracture.

The FRAX tool is not designed to examine the risk of asymptomatic VFs; however we performed this analysis because of the importance of this kind of fractures in the outcome of osteoporosis. It is now well established that identification of VFs change the patient’s diagnostic classification, estimation of fracture risk, and influence the decision for a pharmacological intervention as treatment of patients with prevalent VFs reduces the risk of future fractures even when the baseline T-score is above the osteoporosis diagnostic cutpoint of -2.5. Thus, regarding the important health consequences of osteoporotic VFs, together with the fact that most of them are undiagnosed, emphasizes the need for developing better methods to identify patients with asymptomatic VFs.

Few studies have focused on the predictive value of FRAX risk for identifying women with asymptomatic VFs. In Korea, So et al. in a cross-sectional study including 194 patients found that FRAX underestimated the risk of VFs compared to BMD[24]. Only two studies did a longitudinal analysis. The first one was conducted in 3321 post-menopausal women with low bone mass (60% of them having a femoral neck T score ≤ -2.5) from the FIT (Fracture Intervention Trial) placebo group, of whom 30% had a radiographically detected vertebral fracture at baseline[25]. The AUC was significantly greater for FRAX with femoral neck BMD (AUC =0.71) than FRAX without femoral neck BMD (AUC =0.68; p =0.002). The second study was conducted in a cohort of postmenopausal women (mean age 65.5 years), of whom 12.5% had a radiographically detected VF at baseline and a mean lumbar spine T-score of -0.95, FRAX risk with and without BMD discriminated patients with incident radiographic VFs (FRAX risk with and without BMD predicted VFs with an AUC of 0.66 and 0.62 respectively). This study showed that the strongest risk factor of future VFs was the combination of age, femoral neck BMD and the presence of a radiographic VF at baseline[26].

FRAX has been included as a tool for identifying postmenopausal women in recently updated guidelines published by the NOF in the United States[27] and by the National Osteoporosis Guideline Group [NOGG]), in the UK[16, 28]. The NOF recommends using FRAX when the decision to treat or not to treat is uncertain. It is primarily intended for postmenopausal women and men 40 years of age and older who have T-scores between -1.0 and -2.5 and who are not on treatment, and who have not had spine or hip fractures. The recommended threshold for intervention is a 10-year hip fracture probability ≥3% or major fracture (humerus, forearm, hip or clinical vertebral fracture) probability ≥20%. These NOF guidelines are difficult to apply in all countries as they are based on cost-effectiveness that produce 35% prevention rate for 5 years according to frequency, mortality and morbidity in the USA. Recently, the Japanese committee recommended a cut-off value of 15% on FRAX as treatment threshold for major osteoporotic fractures in osteopenic patients as they noted that FRAX underestimated fractures in the Japanese population[29]. The FRAX scores observed in our study were lower than these recommended thresholds: they were likely influenced by the relatively young age of the patients in this group (60.9 yr) and the high BMI (29.8). Therefore, guidelines should be adjusted according to the socioeconomic model of each country.

As VFs are asymptomatic in two thirds of the cases, our study shows that FRAX risk, even without BMD, can discriminate subjects in whom testing BMD and VFA at the same time would be worthy. Approximately 16% of these women (with osteopenia) and 8.5% of women with normal BMD who otherwise may not have been identified as being at greater fracture risk were found to have unappreciated evident VFs (grade 2 and 3). Thus, assessing BMD alone would have underestimate the number of women needing osteoporosis medications (presence of VFs without osteoporosis).

We found that parity, which is not included in the FRAX algorithm, was significantly associated to the presence of VFs, independent of BMD and of the other CRFs. This risk factor may be more specific of early post-menopausal women than of older women. Hence, we assessed whether adding parity to FRAX would improve the ability to identify young postmenopausal women at high risk of fracture. We found that the new score combining parity and FRAX did not significantly improve the sensitivity of FRAX, and did not have a better discriminant value than lumbar spine T-score alone.

The assessment of fracture was carefully conducted using standard procedures of acquisition, and standard reading of all VFAs. All the morphometric assessments were made by an experienced investigator after training sessions. Before diagnosis of fracture, a non-osteoporotic origin was considered for each deformity. The main limitation lies in the procedures used to select subjects, who were all volunteers and ambulatory, and presumably healthier than the general population which also probably explain the low FRAX scores observed in this study. The Rabat population may not be adequately representative of the whole population. However, since the population living in the area of Rabat is a balanced mixture of the various regions constitutive of the country, we believe the impact on prevalence estimate is limited.

## Conclusions

We showed that, in a cohort of low risk Moroccan postmenopausal women recruited from the general population, FRAX risk without BMD can identify those at highest risk of prevalent asymptomatic osteoporotic VFs suggesting its usefulness as an easy-to-use screening tool in selecting women for VFA indication.

## Electronic supplementary material

Additional file 1:STROBE Statement including the checklist of items that should be included in reports of observational studies.(DOC 89 KB)

Below are the links to the authors’ original submitted files for images.

Authors’ original file for figure 1

## Abbreviations

DXA: Dual-energy x-ray absorptiometry
CRFs: Clinical risk factors
VFs: Vertebral fractures
VFA: Vertebral fracture assessment
BMD: Bone mineral density
BMI: Body mass index
AUC: Areas under the roc curve.
