# Supplementary figures and images for: The discriminative ability of FRAX, the WHO algorithm, to identify women with prevalent asymptomatic vertebral fractures: a cross-sectional study
Source: BMC Musculoskelet Disord. 2014 Nov 4;15:365. doi: 10.1186/1471-2474-15-365 (PMC4226884; doi:10.1186/1471-2474-15-365)

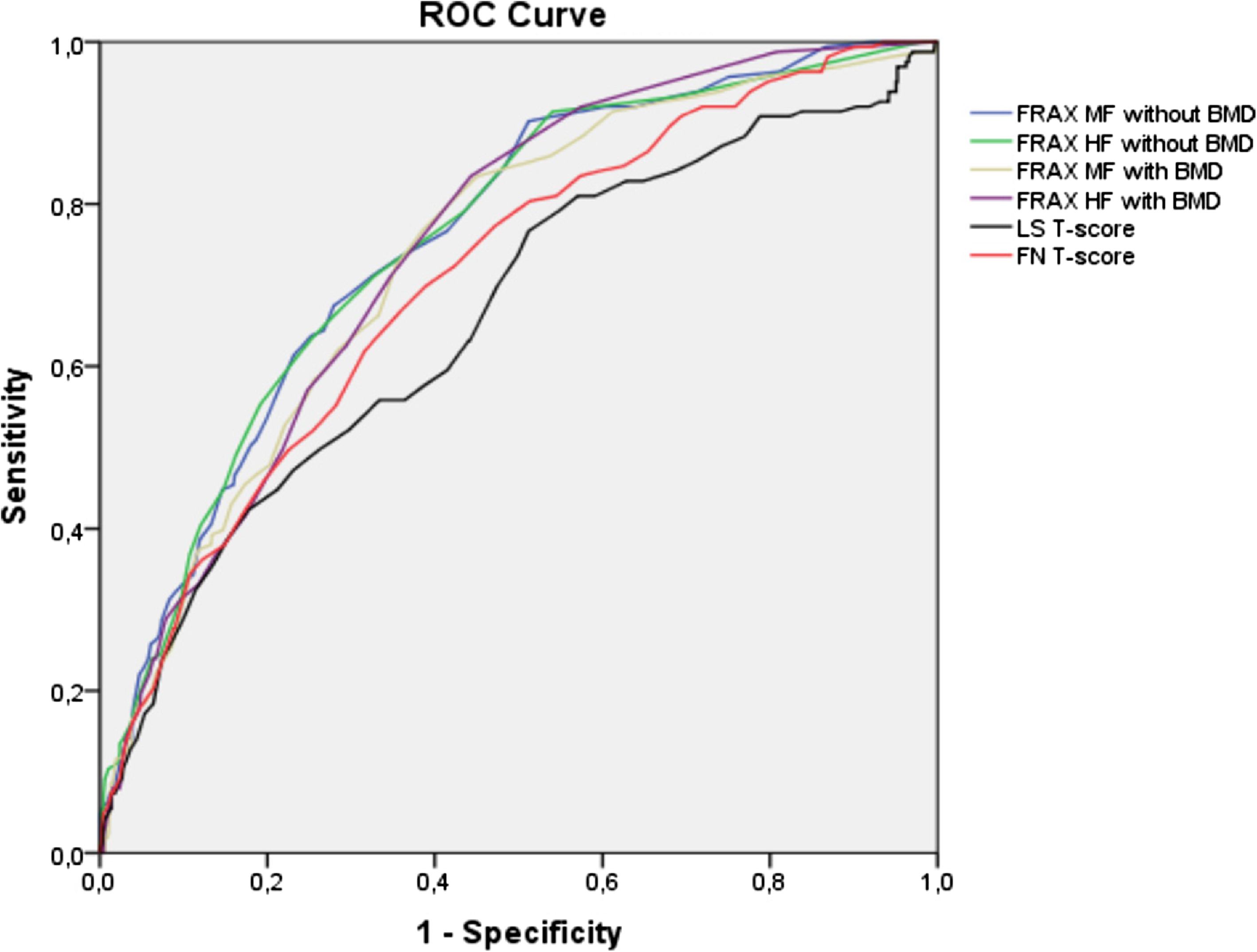

Supplement: Supplementary file 2 — Authors’ original file for figure 1 [file 12891_2014_2297_MOESM2_ESM.tif]
